# Systematic review finds that study data not published in full text articles have unclear impact on meta-analyses results in medical research

**DOI:** 10.1371/journal.pone.0176210

**Published:** 2017-04-25

**Authors:** Christine M. Schmucker, Anette Blümle, Lisa K. Schell, Guido Schwarzer, Patrick Oeller, Laura Cabrera, Erik von Elm, Matthias Briel, Joerg J. Meerpohl, on behalf of the OPEN consortium

**Affiliations:** 1 Cochrane Germany, Medical Center University of Freiburg, Faculty of Medicine, University of Freiburg, Germany; 2 Institute for Quality and Efficiency in Health Care, Cologne, Germany; 3 Institute for Medical Biometry and Statistics, Faculty of Medicine and Medical Center University of Freiburg, Germany; 4 Cochrane Switzerland, Institute of Social and Preventive Medicine (IUMSP), University Hospital Lausanne, Lausanne, Switzerland; 5 Basel Institute for Clinical Epidemiology and Biostatistics, Department of Clinical Research, University of Basel and University Hospital Basel, Switzerland; Universitat Wien, AUSTRIA

## Abstract

**Background:**

A meta-analysis as part of a systematic review aims to provide a thorough, comprehensive and unbiased statistical summary of data from the literature. However, relevant study results could be missing from a meta-analysis because of selective publication and inadequate dissemination. If missing outcome data differ systematically from published ones, a meta-analysis will be biased with an inaccurate assessment of the intervention effect. As part of the EU-funded OPEN project (www.open-project.eu) we conducted a systematic review that assessed whether the inclusion of data that were not published at all and/or published only in the grey literature influences pooled effect estimates in meta-analyses and leads to different interpretation.

**Methods and findings:**

Systematic review of published literature (methodological research projects). Four bibliographic databases were searched up to February 2016 without restriction of publication year or language. Methodological research projects were considered eligible for inclusion if they reviewed a cohort of meta-analyses which *(i)* compared pooled effect estimates of meta-analyses of health care interventions according to publication status of data or *(ii)* examined whether the inclusion of unpublished or grey literature data impacts the result of a meta-analysis.

Seven methodological research projects including 187 meta-analyses comparing pooled treatment effect estimates according to different publication status were identified. Two research projects showed that published data showed larger pooled treatment effects in favour of the intervention than unpublished or grey literature data (Ratio of ORs 1.15, 95% CI 1.04–1.28 and 1.34, 95% CI 1.09–1.66). In the remaining research projects pooled effect estimates and/or overall findings were not significantly changed by the inclusion of unpublished and/or grey literature data. The precision of the pooled estimate was increased with narrower 95% confidence interval.

**Conclusions:**

Although we may anticipate that systematic reviews and meta-analyses not including unpublished or grey literature study results are likely to overestimate the treatment effects, current empirical research shows that this is only the case in a minority of reviews. Therefore, currently, a meta-analyst should particularly consider time, effort and costs when adding such data to their analysis. Future research is needed to identify which reviews may benefit most from including unpublished or grey data.

## Introduction

A meta-analysis as part of a systematic review aims to provide a thorough, comprehensive and unbiased statistical summary of data from the literature.[1] However, relevant study-results could be missing from a meta-analysis because of selective publication and inadequate dissemination (non-dissemination or insufficient dissemination). Even the most comprehensive searches are likely to miss study data which are not published at all such as supplemental unpublished data related to published trials, data obtained from the Food and Drug Administration (FDA) or other regulatory websites or postmarketing analyses hidden from the public. In addition, study data that are not published in conventional journals and, therefore, are not indexed in electronic databases are also likely to be not identified. This so called ‘grey literature’ is not controlled by commercial or academic publishers. It includes non-indexed conference abstracts frequently published in journal collections, dissertations, press releases, government reports, policy documents, book chapters or data obtained from trial registers (Table 1). If the results from missing study data (unpublished and/or study data published in the grey literature) differ systematically from the published data available, a meta-analysis may become biased with an inaccurate assessment of the intervention effect.[2–4]

**Table 1:** Definitions of unpublished, grey and published study data.

**Unpublished data**	not published at all such as supplemental unpublished data related to published trials, data obtained from the Food and Drug Administration (FDA) or other regulatory websites or postmarketing analyses hidden from the public
**Grey literature data**	print or electronic information not controlled by commercial or academic publishers including non-indexed conference abstracts frequently published in journal collections, dissertations, press releases, government reports, policy documents, book chapters or data obtained from trial registers
**Published study data**	published as journal article (usually indexed in electronic database)

There is some evidence that indicates that published randomized controlled trials tend to be larger and show an overall greater treatment effect in favor of the intervention than grey literature trials or unpublished data.[5–8] However, the identification of relevant unpublished study data or data published in the grey literature and their inclusion in meta-analyses can be particularly challenging regarding excessive time, effort and costs. There is also some controversy regarding whether unpublished study data and data published in the grey literature should be included in meta-analyses at all, because they are generally not peer reviewed and their internal validity (risk of bias) may be difficult to assess due to poor reporting of the trials. On the other hand, particularly conference proceedings may take a separate role in the grey literature as they often provide preliminary results or results following intermediate follow-up. A publication by Cook and colleagues showed that 78% of authors of meta-analyses felt that unpublished studies should be included in meta-analyses compared to only 47% of journal editors.[9] Therefore, research is needed to assess the potential impact of inclusion of ‘grey literature’ study data and unpublished data in meta-analyses of health care interventions.

We investigated the impact of study data that were not published in full text articles in scientific journals on pooled effect estimates and the overall interpretation of meta-analyses.

The current review was part of the EU-funded OPEN project (To Overcome failure to Publish nEgative fiNdings; www.open-project.eu) which aimed to investigate non-publication of study data and related dissemination bias through a series of systematic reviews[10–14] following a protocol published previously.[15]

## Methods

### Systematic literature search

We initially searched Medline (Ovid), Embase (Ovid), The Cochrane Library and Web of Science from inception until February 2012. An update search was performed in February 2016. The search strategy was based on combinations of medical subject headings (MeSH) and keywords and was not restricted to specific languages or years of publication. The search strategy used in Medline (Ovid) is presented in S1 Search Strategy. Search strategies for other databases were modified to meet the requirements of each database. The searches were supplemented by checking the bibliographies of any eligible articles for additional references.

### Patient involvement

This research is based on empirical work. Therefore, there was no patient involvement in this methodological systematic review of reviews (so called *umbrella review*).

### Study selection

Titles and abstracts were reviewed using pre-defined inclusion criteria. Full papers of all methodological research projects which included a cohort of meta-analyses (i.e., more than one meta-analysis) and *(i)* compared pooled effect estimates of meta-analyses of health care interventions according to publication status (i.e., published vs. unpublished and/or grey study data) and/or *(ii)* examined whether the inclusion of unpublished and/or grey study data impact the overall findings of a meta-analysis (i.e., from negatively significant to positively significant; from not clinically relevant to clinically relevant) were obtained for detailed evaluation.

All stages of study selection, data extraction and quality assessment were done independently by two reviewers (study selection and data extraction: PO and LC, quality assessment: CS and LKS). Any disagreement during the selection, extraction, and assessment process were resolved by discussion and consensus or with help of a third reviewer (JJM).

We considered a study ‘published’ when it appeared in a peer-reviewed journal. The definition of unpublished and/or grey literature study data had to be in accordance with the definition of ‘unpublished studies’ and ‘grey literature’ described above (see Introduction).

A meta-analysis was defined as mathematical calculation of a weighted summary estimate of a treatment effect by pooling results of two or more studies.

### Outcomes

First, we focused on the extent to which the pooled effect estimate in a meta-analysis changes with the inclusion of unpublished and/or study data published in the grey literature in comparison to published study data. Where possible, we calculated as our primary study outcome a ratio of risk ratios (RRR) or odds ratios (ROR) between the results of published data and the results of unpublished and/or grey literature data and estimate the percentage change (pooled risk ratio from published data divided by pooled risk ratio from unpublished data and/or grey literature data).[15] Thereby, a ratio greater than 1.0 would indicate that published study data showed a greater treatment effect; likewise a ratio below 1.0 would indicate that published data would show a smaller treatment effect. We also intended to calculate a single weighted pooled RRR or ROR to combine ratios from the different methodological research projects to estimate an overall pooled effect, which also takes into account factors such as number of studies, patients and events. For the intended analyses (to calculate a ratio of risk or odds ratios (RRR, ROR) between the results of published study data and unpublished and/or grey study data), the single effect estimates (RR, OR) estimated by the included meta-analyses would be the ‘unit of analyses’.

Second, we aimed to investigate the impact of the inclusion of unpublished or grey literature study data on the interpretation of meta-analyses. This impact can be estimated by calculating the proportion of meta-analyses which showed a change in their interpretation (e.g., from negatively significant to positively significant; from not clinically relevant to clinically relevant).[15]

### Data extraction

We extracted main characteristics of *(i)* the methodological research projects (e.g., baseline data, area of health care, number of meta-analyses included); *(ii)* the meta-analyses (e.g., purpose and scope of meta-analyses, number of studies and participants included); and *(iii)* the studies included in meta-analyses (e.g., number of studies depending on publication status). For more detail see our published protocol.[15]

### Assessment of risk of bias and generalizability of results

No quality assessment tool exists for these types of methodological research projects. Risk of bias (internal validity) and generalizability (external validity) were therefore assessed according to pre-defined criteria which were developed considering empirical evidence on dissemination bias[10, 16] and internal discussion.[15] The assessment of risk of bias was based on *(i)* the selection process; i.e., whether and to which extent the search criteria were reported to identify unpublished and/or grey and published study data; *(ii)* definition of the publication status; i.e., whether explicit criteria were reported for the definition of unpublished or grey literature and published data; *(iii)* role of confounding factors; i.e., whether the difference of the results between unpublished/grey and published study data may be explained by differences in study designs, type of participants or intervention characteristics and not by a true difference in the results between unpublished/grey literature and published data; therefore we investigated whether analyses were stratified or results adjusted for possible confounders. In addition, we investigated the reliability of the data extraction process; i.e., whether data extraction was performed by two researchers independently. Generalizability assessment was based on *(i)* the status of the sample of meta-analyses included; i.e., whether a random, consecutive or selected sample was included and *(ii)* whether the research project selected a broad-ranging sample of meta-analyses that presents the current literature in the field of interest (e.g., in terms of size or diversity of topic).

For data extraction and risk of bias assessment, we relied on information provided in publications of the methodological research projects.

### Statistical analysis and data synthesis

The sparse data did not allow us to apply the predefined statistical analyses neither for the main analysis nor the subgroup analyses.[15] Instead, results of this systematic review are presented descriptively using text and tables.

## Results

### Literature search and selection process

**Fig 1:**
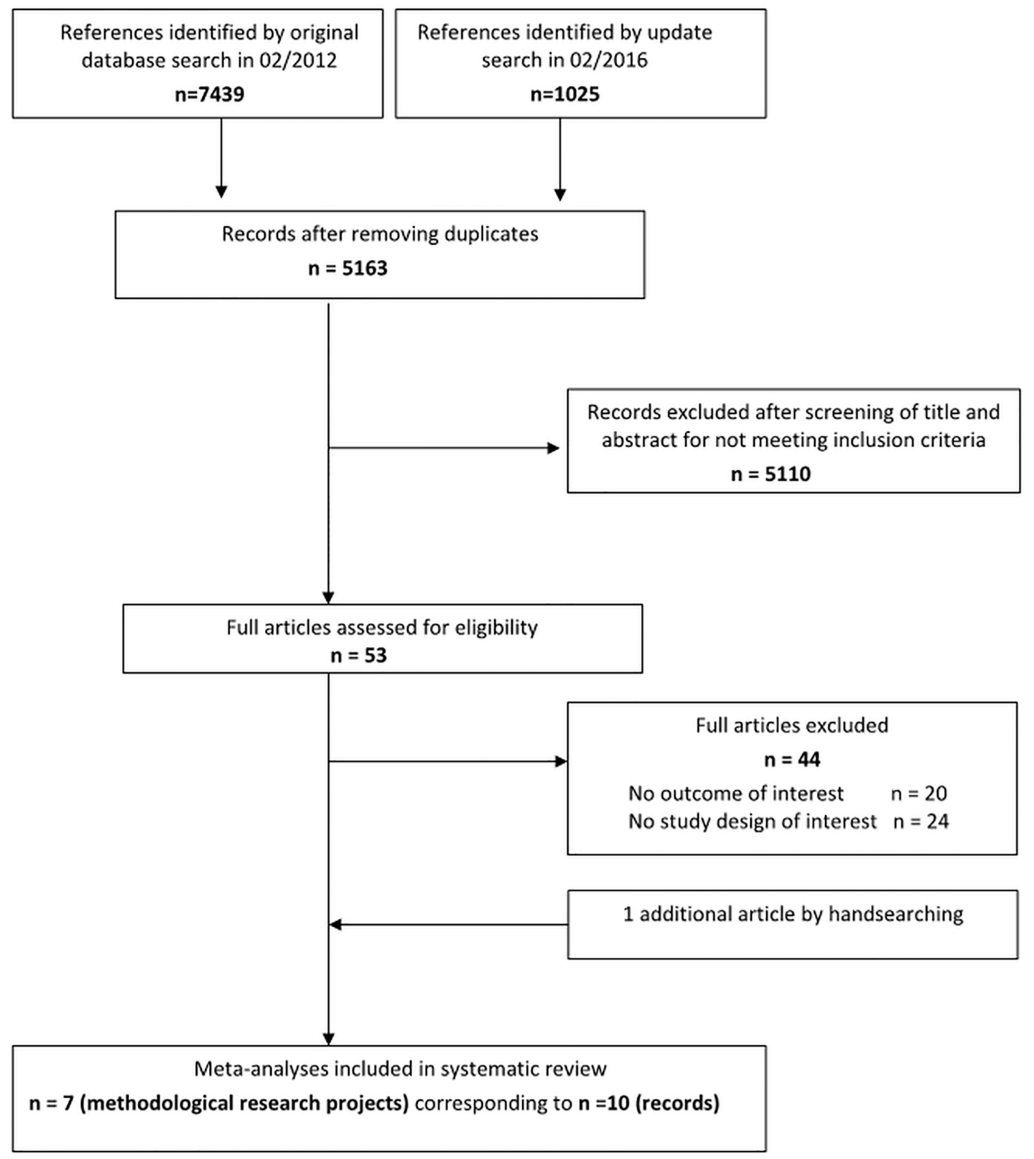
PRISMA statement flow diagram. (as published by *Moher D et al in BMJ 2009;339:b2535*).

The searches identified 8464 citations, including 3301 duplicates (Fig 1). Among the 5163 unique references screened, 10 references[3, 17–25] corresponding to 7 methodological research projects[3, 19–24] were eligible for inclusion in this systematic review.

### Characteristics of included research projects

Main characteristics of the 7 research projects are presented in Tables 2 and 3. In brief, 5 research projects included conventional intervention reviews[3, 20–23], 1 research project was solely based on safety aspects,[24] while another research project included individual participant data meta-analyses.[19] Different medical specialties were displayed in 4 research projects[3, 22–24], while 3 focused on a single medical field.[19–21] In total, 187 meta-analyses with 1617 primary studies (373 unpublished/grey literature studies and 1244 published studies) enrolling a total of 428762 participants (58786 participants in unpublished/grey literature studies and 369976 in published studies) were included. It has to be taken into account that the given numbers of included studies and participants are underestimated because Hart et al[23] and Golder et al[24] did not provide these study characteristics in detail. The publication dates of the latest meta-analyses included in the research projects ranged between 1995[3] and 2014.[24]

**Table 2:** Main characteristics of the included methodological research projects.

	Medical field	Scope of the research	publication date of latest meta-analyses included	N meta-analyses	N studies (median [range])	N participants
**Burdett 2003**[19]	Oncology	Multiple individual participant data meta-analyses	1998	11	120 RCTs (11 [5–19])	18377
**Egger 2003**[22]	Different medical specialties	Multiple meta-analyses on therapeutic or preventive interventions that combined binary outcomes of at least 5 trials	1998	60	783 RCTs (NR [5–53])	167733
**Fergusson 2000**[21]	Surgery	Meta-analyses from a project undertaken by the ‘International Study of Perioperative Transfusion (ISPOT)’ group were used to evaluate different technologies to reduce perioperative red blood cell transfusions	1996	10	114 RCTs (8.5 [2–45])	11142
**Golder 2016**[24]	Adverse effects of different drugs (antidepressants [80%], cardiovascular treatments [20%])	Safety reviews	2014	20*	99 non-RCTs^#^ (7.5 [1–28])	>7942
**Hart 2012**[23]	Drug trials of new molecular entities approved by the FDA between 2001 and 2002	Reanalysis of published meta-analyses including unpublished FDA data (efficacy outcomes, N = 41; safety outcome, N = 1)	2010	42**	NR	NR
**Martin 2005**[20]	Psychiatry	Reanalysis of Cochrane reviews including only published studies on atypical vs typical antipsychotic drugs for schizophrenia (outcome: ‘leaving study early for any reason’)	2004	3	34 RCTs (NR [5–23])	6141
**McAuley 2000**[3]	Different medical specialties	Multiple meta-analyses drawn randomly from an existing database of meta-analyses	1995	41	467 RCTs (10 [6–19])	217427

* 20 meta-analyses corresponding to 11 systematic reviews. Golder et al 2016[24] included 28 meta-analyses in total; from 20 meta-analyses pooled estimates of published studies alone could be compared against unpublished and published estimates.

** 42 meta-analyses corresponding to 9 systematic reviews (for 9 drugs across 6 drug classes).

^#^ The number of studies was reported in the previous review from Golder and colleagues in 2010[25] evaluating only 5 meta-analyses. In the up-dated version of the review (Golder et al 2016[24]), the number of included studies could not be derived. Therefore the number of included studies is underestimated.

FDA: Food and Drug Administration, N: total number, NR: not reported, NSAID: non-steroidal anti-inflammatory drugs, RCTs: randomized controlled trials.

**Table 3:** Main characteristics of studies included in analyses.

	Number Studies				Number Participants			
	grey literature (median [range])	unpublished (median [range])	combined grey literature and unpublished studies (median [range])	published (median [range])	grey literature (median [range])	unpublished (median [range])	combined grey literature and unpublished studies (median [range])	published (median [range])
**Burdett 2003**[19]	21 (NR)	24 (NR)	45 (NR)	75 (NR [3–15])	2924 (NR)	3297 (NR)	6221 (NR)	12156 (NR [198–2899])
**Egger 2003**[22]	NR	NR	153 (NR)	630 (NR)	NR	NR	21573 (91 [9–1012])	146160 (102 [8–5042])
**Fergusson 2000**[21]	5 (NR)	1 (NR)	6 (NR)	108 (NR)	NR	NR	820 (149 [85–250])	10322 (683 [240–828])
**Golder 2016**[24]	-	52 (8 [1–28])	52 (8 [1–28])	47 (7 [1–22])	-	>4590 (NR)	>4590 (NR)	>3352 (NR)
**Hart 2012**[23]	-	NR	-	NR	-	NR*	-	NR
**Martin 2005**[20]	15* (NR [4–8])	-	15** (NR [4–8])	19*** (NR [1–15])	2296^#^ (NR)	NR	2296^#^ (NR)	3845^##^ (NR)
**McAuley 2000**[3]	NR	NR	102 (2 [1–3])	365 (NR)	NR	NR	23286 (84 [48–190])	194141 (113 [59–228])

* The proportion (calculated as number of participants from unpublished outcomes divided by total number of participants) of unpublished FDA data in meta-analysis ranged between 8 and 94%.

** Three meta-analyses: meta-analysis I: N = 4 studies, meta-analysis II: N = 3 studies, meta-analysis III: N = 8 studies.

*** Three meta-analyses: meta-analysis I: N = 1 studies, meta-analysis II: N = 3 studies, meta-analysis III: N = 15 studies.

^#^ Three meta-analyses: meta-analysis I: N = 179 participants, meta-analysis II: N = 665 participants, meta-analysis III: N = 1452 participants.

^##^ Three meta-analyses: meta-analysis I: N = 60 participants, meta-analysis II: N = 959 participants, meta-analysis III: N = 2826 participants.

NR: not reported, -: grey literature was not included in this research project, only unpublished data.

### Assessment of risk of bias and generalizability of results

Table 4 presents the assessment of risk of bias and generalizability of results for each research project. Regarding risk of bias, each research project reported how unpublished or grey literature study data were identified within meta-analyses. Unpublished or grey literature data (e.g., in terms of conference abstracts, dissertations or editorials) were sufficiently defined in all research projects. The main limitation of the research projects was that most of them (except for Golder et al[24]) did not allow us to judge whether grey literature or unpublished study data in comparison to published data were adequately matched (e.g., in terms of study aim or sample size) or adjusted for confounders.

**Table 4:** Risk of bias and generalizability of included methodological research projects.

	Risk of Bias*				Generalizability*	
	Appropriate methodology given to identify grey/unpublished and published studies?	Appropriate definition of grey and/or unpublished studies given?	Appropriate adjustment for confounders between grey/unpublished and published studies?	Reliability of the data extraction process?	Appropriate sampling method (e.g., random sample of meta-analyses)?	Appropriate presentation of the medical field of interest?
**Burdett 2003**[19]	+	+	?	?	-	-
**Egger 2003**[22]	+	+	?	+	+	+
**Fergusson 2000**[21]	+	+	?	+	+	+
**Golder 2016**[24]	+	+	+	+	+	-
**Hart 2012**[23]	+	+	?	+	+	+
**Martin 2005**[20]	+	+	?	?	-	?
**McAuley 2000**[3]	+	+	?	?	?	-

* For the assessment of risk of bias and generalizability of the results, we relied on the reporting of the methodological research project.

***Risk of bias***

**Methodology to identify unpublished/grey studies: +** at least one indicator of appropriate searches for unpublished/grey studies was given (e.g., searches of conference proceedings, contacts with authors, companies that produced the therapies and/or co-investigators); **-** no indicators were reported;**?** not enough information for a judgment.

**Definition of grey/unpublished studies: +** detailed definition of grey literature (e.g., abstracts, book chapters) and/or unpublished data (e.g., studies as part of a new drug application, FDA data) is given that is compatible with our definition used for this review; **-** grey and/or unpublished literature was not predefined;**?** not enough information for a judgment.

**Adjustment for potential confounders: +** the groups (unpublished or grey literature studies vs published studies) are matched in terms of e.g., study aim, study population, dosage and study quality *or*, if not, adjustment for confounding factors by multivariable analysis was carried out; **-** no (adequate) analyses were carried out or study groups were clearly not comparable; ? not enough information for a judgment.

**Data extraction: +** data extraction was carried out by 2 researchers independently; **-** data extraction was carried out by 1 researcher;**?** not enough information for a judgement.

***Generalizability***

**Sampling method (how was the sample determined?): +** a random or consecutive sample was used, or all meta-analyses within a predefined period of time were selected; **-** a selected sample of meta-analyses was used;**?** sampling method not reported.

**Re-presentation of the medical field of interest: +** the sample of meta-analyses re-presents the medical field of interest, e.g., a broad-ranging sample was used; **-** the selected sample was not broad enough to reflect the current literature;**?** not enough information for a judgement.

Generalizability of results was low or unclear in four research projects.[3, 19, 20, 24] It means that the results of these research projects were either based on a selected sample of meta-analyses (e.g., meta-analyses from one research group only were used) or the medical field of interest was not sufficiently represented (e.g., only few rare sorts of cancers or a small range of interventions were considered).

### Effect of unpublished or grey literature study data on pooled estimates in meta-analyses

The effects of unpublished or grey literature studies on pooled estimates in meta-analyses are shown in Table 5. One research project (including 467 randomized controlled trials) showed that published studies had a larger pooled treatment effect in favor of the intervention than unpublished studies (ROR 1.15, 95% CI 1.04–1.28).[3] In the remaining research projects pooled effect estimates were not significantly changed by the inclusion of unpublished or grey literature data. However, Egger et al[22] presented the pooled effect estimate across different medical specialties (ROR 1.07, 95% CI 0.98; 1.15)–but also separated effect estimates for selected medical fields. In the field of obstetrics and gynaecology this pooled analysis showed that published results are more positive than unpublished results (ROR 1.34, 95% CI 1.09–1.66). In psychiatry there was a similar trend but pooled estimates did not reach statistical significance (ROR 1.61, 95% CI 0.9–2.9). The combination of estimates across methodological research projects was not possible due to differences in the definitions of effect estimates (some research projects reported hazard ratios, other odds ratios or risk ratios, or even weighted mean differences) and clinical heterogeneity (different aims of the research projects regarding safety and efficacy outcomes).

**Table 5:** Outcomes of the included methodological research projects.

	Ratio of effect estimates	Published studies (95% CI)	Overall studies (95% CI)	Published *vs* unpublished and/or grey literature studies (95% CI)	Impact of unpublished or grey literature studies on the interpretation of meta-analyses
**Burdett 2003**[19]	HR	0.93 (0.90; 0.97)	0.96 (0.93; 0.99)	-	-
**Egger 2003**[22]	OR (efficacy)	-	-	1.07 (0.98; 1.15)^#^	(i) “unpublished trials show less benefical effects than published trials”; (ii) “removal of the grey literature from the sample of meta-analyses resulted in a change in pooled estimates from a 28% decrease to a 24% increase in benefit”; (iii) “in 72% of the 60 meta-analyses the percentage changes were <5%”; (iv) “in meta-analyses in which the change of the pooled estimate was 5% or more, 8 showed increased and ten showed decreased benefit”; (v) “average precision of pooled estimates decreased from 9.22 to 8.41 with the removal of grey trails”; (vi) “3 meta-analyses lost and one gained statistical significance at the 5% level”
				1.34 (1.09; 1.66)^##^	
				1.61 (0.90; 2.90)^##^	
**Fergusson 2000**[21]	OR* (efficacy)	0.57 (0.38; 0.86)	0.50 (0.34; 0.76)	-	“odds ratios of the meta-analyses were not substantially changed by their inclusion”
**Golder 2016**[24]	RR** (safety)	-	-	0.95 (0.65; 1.37)°	(i) “inclusion of unpublished data increased the precision of the pooled estimates (narrower 95% confidence intervals) in 15 of the 20 meta-analyses”; (ii) “there were 3 analyses where a non statistically significant pooled estimate of increased risk (published studies alone) became statistically significant after unpublished data was added”; (iii) “there was 1 meta-analyses in which a significantly increased risk estimate from published studies was rendered non significant after addition of unpublished data”; (iv) “the direction and magnitude of the difference varies and is not consistent”
**Hart 2012**[23]	RR, OR, WMD	34 meta-analyses: statistically significant in favour of the drug^§^	38 meta-analyses: statistically significant in favour of the drug^§^	-	(i) “addition of unpublished data varied by drug and outcome”; (ii) “addition of unpublished data caused in 46% lower, in 7% identical, in 46% greater effect estimates than published data”
	RR, OR, WMD	7 meta-analyses: no difference between the interventions^§^	3 meta-analyses: no difference between the groups^§^		
	RR	0.85 (0.71;1.03)	0.92 (0.82; 1.02)	-	“addition of unpublished data showed more harm”
**Martin 2005**[20]	RR (efficacy)	0.69 (0.35; 1.37)	0.69 (0.51; 0.94)	-	-
	RR (efficacy)	0.87 (0.73; 1.02)	0.87 (0.76; 0.99)		
	RR (efficacy)	0.67 (0.56; 0.82)	0.69 (0.59; 0.80)		
**McAuley 2000**[3]	OR (efficacy)	-	-	1.15 (1.04; 1.28) / 1.02 (0.91; 1.14)^§§^	(i) “removal of the grey literature changed the estimate of intervention effectiveness by 10% or more (in 14 out of 41 meta-analyses)”; (ii) “in 9 of these 14 cases, removal resulted in the intervention effect moving away from unity”; (iii)“in 3 meta-analyses, the exclusion of the grey literature resulted in a change in the significance of the results, from non-significant to significant, in 2 cases”

* Only the effect estimate for the intervention ‘tranexamic acid’ to reduce perioperative red blood cell transfusions was provided. For other interventions it was reported that the odds ratios of the meta-analyses were not substantially changed by inclusion of grey (non-peer reviewed) reports.

** Ratio of risk ratios for common adverse effects.

^#^ Effect estimate across all medical specialties.

^##^ Egger et al 2003 also separated effect estimates for selected medical fields. In obstetrics and gynaecology this pooled analysis showed that published results are more positive than unpublished results (ROR: 1.34 [1.09–1.66]). In psychiatry there was a similar trend but pooled estimates did not reach statistical significance (ROD: 1.61 [0.90–2.90]).

^§^ Individual data of summary statistic are presented in Table 2 of the original publication of Hart and colleagues in 2012.[23]

^§**§**^ Comparison of abstracts and full publications.

° The pooled effect estimate was only given in the previous review from Golder and colleagues in 2010.[25] This risk estimates was derived from only 5 meta-analyses. In the research project from 2016,[24] a pooled risk estimate over all meta-analyses was not provided.

OR: odds ratio; RR: relative risk, WMD: weighted mean difference.

### Impact of unpublished or grey literature study data on the interpretation of meta-analyses

Five research projects provided additional information on the overall impact of unpublished or grey literature study data on the interpretation of the results. The results are descriptively summarized in Table 5. Hart and colleagues[23] reported that the addition of unpublished data to their sample of meta-analyses caused in 46% lower, in 7% identical and in 46% greater effect estimates than published data. In the research project from Egger et al[22] removal of grey literature data resulted in a change in pooled estimates from a 28% decrease to a 24% increase in benefit. McAuley and colleagues[3] reported that removal of grey literature data changed the estimate by at least 10%. Thereby, significance of the results was affected in 3 out of 41 meta-analyses.

On the other hand, Fergusson and colleagues[21] and Golder and colleagues[24] stated that ‘effect estimates were not substantially changed’[21] or that ‘the direction and magnitude of the difference varies and is not consistent’[24] when unpublished or grey literature data are added.

## Discussion

### Principal findings

Although it was shown that some case samples of meta-analyses not including grey literature or unpublished data clearly overestimate treatment effects,[6–8] quantifying this effect by considering all meta-epidemiological studies (so called *methodological research projects*) reveals that this affects only a minority of reviews. In the majority of meta-analyses over a wide range of medical fields, excluding unpublished trials had no or only a small effect on the pooled estimates of treatment effects. However, in some instances more substantial, statistically significant changes were observed (overestimating the effect between 9 and 60%)[22] There may be a tendency in research areas involving new drugs or technologies to publish the most exciting and positive results more rapidly, and negative ones less quickly, if at all.[10] Also sponsorship of drug and device studies by the manufacturer leads to more favourable results and conclusions than sponsorship by other sources.[26] Consequently, the problem of dissemination bias could be more pronounced in medical areas in which relevant innovations are being developed at quick pace or when trials are published close to drug approval. This assumption, however, could not be proven with the available empirical data.

Our research and other reviews[5, 27] revealed that unpublished trials are often smaller (e.g., Table 3, differences in medians between unpublished or grey literature study data and published data: 11,[22] 534,[21] and 29[3] patients, respectively). Small sample sizes may be one of the reasons that unpublished or grey literature study data are less likely to produce statistically significant results than published data. However, if study size was the only factor impacting on the likelihood of publication this would not result in bias, but a lack of precision with wider confidence intervals of effect estimates.

Methodological research projects included in this review used different statistical methods to determine the contribution of unpublished data in meta-analyses. For example, Egger *and colleagues[22]* used the statistic chosen by the original reviewers of the meta-analyses to calculate pooled effect estimates separately for unpublished and published trials. Thereafter, weighted averages for all these ratios were calculated using random effects models. McAuley *and colleagues*[3] chose a fixed effect logistic regression model which requires individual patient data from each trial. This approach ignores heterogeneity between trials and between meta-analyses. In general, too little consideration has been given to appropriate statistical methods for this type of meta-epidemiological research so far. This may lead to an underestimation of the uncertainty of effect estimates due to unpublished data in meta-analyses.[28]

None of the methodological research projects addressed the problem of multiple journal publications.[29] Unaccounted duplicate publication may inflate the number of participants and/or events leading to increased precision and, obviously, causes dissemination bias.

Evidence from a Cochrane review has shown that only about half of all trials reported as abstracts and presented at conferences are subsequently published in full.[16] In addition, it takes, on average, three years for a trial reported as an abstract to be eventually published in a peer-reviewed journal. Therefore, excluding them seems an arbitrary act that may bias the results. On the other hand, McAuley *and colleagues* showed that the inclusion of abstracts had no relevant impact on pooled estimates of meta-analyses over different medical fields.[3] Moreover, concerns have been raised regarding the methodological and reporting quality in unpublished studies, because grey or unpublished literature is often not peer reviewed. We believe that abstracts may take a separate role in the grey literature as they often provide preliminary study results, results following intermediate follow-up, or unexpected findings. Consequently, when a researcher decides to include unpublished or grey literature study data in meta-analyses, it is important to run sensitivity analyses to identify possible differences between results from unpublished or grey literature studies and from fully published papers. While there is no doubt that studies that have positive results are subsequently published as full-length journal articles more often than studies with negative results,[10] lack of time of the authors may be a major reason for non-publication of research—independent of the direction of results.[30]

### Strengths and weaknesses of this review

This systematic review sought to comprehensively synthesize the body of research on the impact of including unpublished study data and data published in the grey literature in meta-analyses. By discussing multiple study characteristics and potential confounders related to unpublished studies and studies published in the grey literature, we could not identify sufficient evidence to conclude whether or to which extent inclusion of unpublished and grey study data have an impact on the pooled effect estimates and the conclusions from meta-analyses. Nevertheless, the available research projects demonstrates that availability of unpublished and grey literature data leads to a more precise risk estimates with narrower 95% confidence intervals, thus representing higher evidence strength according to the GRADE evaluation (Grades of Recommendation, Assessment, Development and Evaluation).[31] In addition, we developed criteria to assess both risk of bias and generalizability for this specific type of empirical research which may be of high value in future methodological research. Our strategy was not focused on the results of single meta-analyses including published and unpublished data, but on meta-epidemiological studies. We expected that theses research projects would allows us to estimate the “average” overestimation of treatment effects due to dissemination bias.

However, we are aware that our findings have several limitations: First, we could not identify sufficient research projects to conclude whether or to which extent inclusion of unpublished and grey study data have an impact on the conclusions from meta-analyses. Second, the risk of bias assessment revealed that the internal validity may be hampered due to the lack of appropriate adjustment for potential confounders between published and unpublished or grey literature data in the identified methodological research projects. Second, our research is mainly limited to selected samples of medical literature (e.g., rare sorts of cancers or a small range of adverse effects), and hence the findings may not be generalizable to other medical fields. However, most medical fields assessed were large and permitted evaluation of a large number of studies.[19, 21] Another weakness of our study relates to its retrospective nature and its reliance on what authors described as comprehensive literature searches. We did not assess whether the sample of trials identified by these authors was in fact complete and whether searches were truly comprehensive. If searches were inadequate, so that many unpublished or grey literature studies with negative results were consciously or unconsciously omitted, then our review may underestimate the impact of dissemination bias. Roughly the same would be true, if predominantly unpublished or grey literature studies with similar results to published studies were identified by inadequate searches. But we could not judge how often this happened. On the other hand, we are concerned about the possibility of dissemination bias (in particular reporting bias), where investigators may have chosen not to write up their results (e.g., for a subgroup of patients) if they did not find any significant differences between published and unpublished study data. We believe that the impact of unpublished or grey literature data on pooled estimates could be assessed more thoroughly if the intention to compare data sources according to publication status was built in at the protocol stage of these meta-analyses.

Time, effort, and cost involved in locating and retrieving unpublished data and grey literature makes its inclusion in reviews challenging. Legal obligation to prospectively register trials and make results available after completion of the trial in many countries (including the United States and Europe), different registries for clinical trials such as the International Clinical Trials Registry Platform (ICTRP) or the database ClinicalTrials.gov, internet-based grey literature resources, journals devoted to negative trials, or efforts taken by various groups, including Cochrane (through trial registries), may further ease the identification and inclusion of unpublished data in meta-analyses.

We acknowledge that more than half of all published systematic reviews are not including meta-analyses.[32] Despite our focus on the impact of unpublished and grey literature study data on pooled effect estimates in meta-analyses, we believe that our findings are also applicable to systematic reviews with qualitative/descriptive summaries.

### Comparison with other systematic reviews

We are aware of one methodological Cochrane review which addressed the impact of unpublished and grey literature data in meta-analyses on the basis of meta-epidemiological studies.[5] This review was published in 2007 and concluded that grey literature trials show an overall greater treatment effect than published trials. The authors acknowledged that the evidence is sparse and that more efforts are needed to identify a complete and unbiased set of trials irrespective of whether they have been published or not. In contrast to our review, this methodological review is nearly 10 years old, did not apply methods to address risk of bias and generalizability of the results of the included studies covering the given research question.

Our findings suggest that dissemination bias is a very serious threat to the results of meta-analyses, but not always impacts their results. This finding is supported by other studies (not meeting the inclusion criteria for this review) based on unpublished FDA data and published data.^e.g.,^ [6, 33] One of these meta-analyses investigating selective publication of antidepressant trials found a bias toward the publication of positive results, resulting in an effect size nearly one third larger than the effect size derived from unpublished FDA data.[6] Controversially, MacLean and collegues[33] reported that risk ratios for dyspepsia did not significantly or clinically differ using published or unpublished FDA data.

### Implications for policy makers and further research

This work has implications for researchers and those who use meta-analyses to help inform clinical and policy decisions. *(i)* Investigators should ensure a comprehensive systematic literature search to avoid or at least attenuate the effect of dissemination bias. Such searches can be resource-intensive particularly when unpublished and grey literature data need to be identified. If the available resources do not permit comprehensive searches to identify unpublished or grey literature data, we strongly recommend (at least) a search in trial registries (such as the ICTRP and ClinicalTrials.gov) and websites of regulatory authorities which is less resource-intensive than searching for conference proceedings or dissertations, contacting experts, the industry and authors. When including unpublished or grey literature data sensitivity analyses should be carried out taking into account that this research may provide only preliminary results, is usually not peer reviewed and/or at higher risk of bias. It is obvious that even a thorough literature search cannot eliminate dissemination bias. Therefore, it is also of great importance to apply additional methods for detecting, quantifying and adjusting for dissemination bias in meta-analyses.[14] Such methods include graphical methods based on funnel plot asymmetry, statistical methods, such as regression tests, selection models, and a great number of more recent statistical approaches. [2] [34–36] However, the empirical research work of Mueller et al 2016 concluded that it remains difficult to advise which method should be used as they are all limited and only few methods have been validated in empirical evaluations using unpublished studies obtained from regulators (e.g., FDA studies).[14] Selective outcome reporting in clinical studies is also an indicator for hidden or missing data, especially when only selective slices of the complete clinical trial are published or when studies show huge drop-out rates without providing reasons for these patients who left the study.[7, 37, 38] Overall, researchers should carefully consider the potential risk of dissemination bias when interpreting their findings. *(ii)* Those using meta-analyses to assist with clinical and policy decisions should also be aware of dissemination bias, because dissemination bias may have direct impact for patient care.[39] *(iii)* Major improvements have been made in the accessibility of data by initiatives such as the AllTrials campaign (www.alltrials.net) calling for all trials to be registered and the methods and results to be reported, the European Medicines Agency (EMA) policy on publication of clinical data on request since 2015, the obligatory release of results in trial registries by the European law (Clinical Trial Regulation), the FDA Amendment Act in 2007 and advocacy from the Cochrane Collaboration to fully implement such policies. Although progress has been made, there are still major issues related to unrestricted data access. Even when data are released, they can be incomplete, selective or not in compliance with the results reported in study registers such as ClinicalTrials.gov.[40] [41] [42] Therefore, further action is required to progress toward unrestricted data access. Particularly the full release of clinical study reports (CSR) may contain more information than other unpublished sources and, therefore, may have the potential to overcome existing problems.[43] *(iv)* Our research indicates that it seems that it will not be possible for a meta-analyst to judge before-hand whether the addition of unpublished and grey literature study data impacts the pooled effect estimates and leads to a change in the overall conclusions. *(v)* Finally, even the most comprehensive search for grey and unpublished data will not allow a final judgment whether the identified sample is in fact complete and representative for all of the hidden data.

## Supporting information

S1 Search Strategy Search strategy for Ovid MEDLINE. (DOCX)
