# Supplementary material for: Systematic review finds that study data not published in full text articles have unclear impact on meta-analyses results in medical research
Source: PLoS One. 2017 Apr 25;12(4):e0176210. doi: 10.1371/journal.pone.0176210 (PMC5404772; doi:10.1371/journal.pone.0176210)
Supplement: S1 Search Strategy — (DOCX) [file pone.0176210.s001.docx]

**Figure S2. Search Strategy for OvidSP MEDLINE.**

1 exp Publishing/sn

2 *publishing/

3 publication bias/

4 selection bias/

5 exp manuscripts as topic/

6 ((data or finding? or information or evidence or study or studies or trial? or paper? or article? or report* or literature or work or manuscript? or abstract* or result?) adj6 (unpublish* or un-publish* or unreport* or un-report* or nonpublish* or non-publish* or nonpublicat* or non-publicat* or (publication? adj3 rate?) or "not publish*")).ti,ab.

7 (underreport* or under-report* or selective report* or selective publish* or selective publicat* or (final* adj2 (report* or publish* or publicat* or manuscript? or paper? or article?)) or (full? adj2 (report* or publish* or publicat* or manuscript? or paper? or article?)) or (subsequent* adj2 (report* or article? or paper? or publi* or manuscript?)) or (sub-sequent* adj2 (report? or article? or paper? or publi* or manuscript?)) or (complete* adj2 (report* or article? or paper? or publish* or publicat* or manuscript?))).ti,ab.

8 (bias* adj3 (publish* or publicat*)).ti,ab.

9 or/1-8

10 exp animals/ not humans/

11 meta-analysis as topic/

12 Guidelines as Topic/ or Practice Guidelines as Topic/

13 exp Clinical Trials as Topic/

14 meta-analysis.pt.

15 (guideline or practice guideline).pt.

16 (guideline? or metaanaly* or meta-analy* or metanaly* or meta-synthe* or metasynthe* or meta-regressi* or metaregressi*).ti,ab.

17 (systematic* adj3 (review* or overview*)).ti,ab.

18 exp Technology Assessment, Biomedical/

19 (health technology assessment? or HTA).ti,ab.

20 or/11-19

21 9 and 20

22 21 not 10

23 ((implication? or impact? or influenc* or effect? or differen*) adj6 (publication bias* or unpublish* or un-publish* or unreport* or un-report* or nonpublish* or non-publish* or nonpublicat* or non-publicat* or "not publish*")).ti,ab.

24 ((implication? or impact? or influenc* or effect? or differen*) adj6 (selective report* or selective publish* or selective publicat* or (final* adj2 (report* or publish* or publicat* or manuscript? or paper? or article?)) or (full? adj2 (report* or publish* or publicat* or manuscript? or paper? or article?)) or (subsequent* adj2 (report* or article? or paper? or publi* or manuscript?)) or (sub-sequent* adj2 (report? or article? or paper? or publi* or manuscript?)) or (complete* adj2 (report* or article? or paper? or publish* or publicat* or manuscript?)))).ti,ab.

25 ((unpublish* or un-publish* or unreport* or un-report* or nonpublish* or non-publish* or nonpublicat* or non-publicat* or "not publish*") adj6 publish*).ti,ab.

26 (underreport* or under-report* or selective report* or selective publish* or selective publicat* or (final* and (report* or publish* or publicat* or manuscript? or paper? or article?)) or (full? and (report* or publish* or publicat* or manuscript? or paper? or article?)) or (subsequent* and (report* or article? or paper? or publi* or manuscript?)) or (sub-sequent* and (report? or article? or paper? or publi* or manuscript?)) or (complete* and (report* or article? or paper? or publish* or publicat* or manuscript?))).ti.

27 (unpublish* or un-publish* or unreport* or un-report* or nonpublish* or non-publish* or nonpublicat* or non-publicat* or "not publish*" or bias*).ti.

28 or/23-27

29 22 and 28

30 22 and (3 or 4)

31 22 and (11 or 12)

32 31 and (6 or 7)

33 *meta-analysis as topic/

34 *Guidelines as Topic/ or *Practice Guidelines as Topic/

35 22 and (33 or 34)

36 (6 or 7) and (3 or 8)

37 29 or 30 or 32 or 35 or 36

38 (unpublish* or un-publish* or unreport* or un-report* or nonpublish* or non-publish* or nonpublicat* or non-publicat* or "not publish*").ti,ab.

39 7 or 38

40 37 and 39

41 11 or 12

42 3 and 41

43 40 or 42

44 43 not 10

45 remove duplicates from 44
